# Three-dimensional visualisation of the fetal heart using prenatal MRI with motion-corrected slice-volume registration: a prospective, single-centre cohort study

**DOI:** 10.1016/S0140-6736(18)32490-5

**Published:** 2019-04-20

**Authors:** David F A Lloyd, Kuberan Pushparajah, John M Simpson, Joshua F P van Amerom, Milou P M van Poppel, Alexander Schulz, Bernard Kainz, Maria Deprez, Maelene Lohezic, Joanna Allsop, Sujeev Mathur, Hannah Bellsham-Revell, Trisha Vigneswaran, Marietta Charakida, Owen Miller, Vita Zidere, Gurleen Sharland, Mary Rutherford, Joseph V Hajnal, Reza Razavi

**Affiliations:** a School of Biomedical Engineering & Imaging Sciences, King's College London, King's Health Partners, St Thomas' Hospital, London, UK; b Department of Congenital Heart Disease, Evelina London Children's Hospital, Guy's and St Thomas' NHS Foundation Trust, London, UK; c Department of Computing (BioMedIA), Imperial College London, London, UK

## Abstract

**Background:**

Two-dimensional (2D) ultrasound echocardiography is the primary technique used to diagnose congenital heart disease before birth. There is, however, a longstanding need for a reliable form of secondary imaging, particularly in cases when more detailed three-dimensional (3D) vascular imaging is required, or when ultrasound windows are of poor diagnostic quality. Fetal MRI, which is well established for other organ systems, is highly susceptible to fetal movement, particularly for 3D imaging. The objective of this study was to investigate the combination of prenatal MRI with novel, motion-corrected 3D image registration software, as an adjunct to fetal echocardiography in the diagnosis of congenital heart disease.

**Methods:**

Pregnant women carrying a fetus with known or suspected congenital heart disease were recruited via a tertiary fetal cardiology unit. After initial validation experiments to assess the general reliability of the approach, MRI data were acquired in 85 consecutive fetuses, as overlapping stacks of 2D images. These images were then processed with a bespoke open-source reconstruction algorithm to produce a super-resolution 3D volume of the fetal thorax. These datasets were assessed with measurement comparison with paired 2D ultrasound, structured anatomical assessment of the 2D and 3D data, and contemporaneous, archived clinical fetal MRI reports, which were compared with postnatal findings after delivery.

**Findings:**

Between Oct 8, 2015, and June 30, 2017, 101 patients were referred for MRI, of whom 85 were eligible and had fetal MRI. The mean gestational age at the time of MRI was 32 weeks (range 24–36). High-resolution (0·50–0·75 mm isotropic) 3D datasets of the fetal thorax were generated in all 85 cases. Vascular measurements showed good overall agreement with 2D echocardiography in 51 cases with paired data (intra-class correlation coefficient 0·78, 95% CI 0·68–0·84), with fetal vascular structures more effectively visualised with 3D MRI than with uncorrected 2D MRI (657 [97%] of 680 anatomical areas identified *vs* 358 [53%] of 680 areas; p<0·0001). When a structure of interest was visualised in both 2D and 3D data (n=358), observers gave a higher diagnostic quality score for 3D data in 321 (90%) of cases, with 37 (10%) scores tied with 2D data, and no lower scores than for 2D data (Wilcoxon signed rank test p<0·0001). Additional anatomical features were described in ten cases, of which all were confirmed postnatally.

**Interpretation:**

Standard fetal MRI with open-source image processing software is a reliable method of generating high-resolution 3D imaging of the fetal vasculature. The 3D volumes produced show good spatial agreement with ultrasound, and significantly improved visualisation and diagnostic quality compared with source 2D MRI data. This freely available combination requires minimal infrastructure, and provides safe, powerful, and highly complementary imaging of the fetal cardiovascular system.

**Funding:**

Wellcome Trust/EPSRC Centre for Medical Engineering, National Institute for Health Research.

## Introduction

The normal physiological changes that accompany the transitional circulation after birth can have profound effects on newborns with congenital heart disease, the most prevalent group of birth defects worldwide. Detecting severe cardiovascular malformations before birth provides an opportunity for potentially life-saving care to be delivered immediately after delivery, improving postnatal outcomes,^1^, ^2^, ^3^, ^4^ and allowing time for parents to process the potential implications for the child and their family.

Throughout most of the past century, understanding of congenital heart disease before birth was limited to post-mortem and animal studies.^5^ The introduction of fetal echocardiography in the 1980s brought about a revolution in in-vivo prenatal diagnosis. Indeed, in most scenarios, ultrasound remains the method of choice because of its ease of use, speed, and high diagnostic accuracy.^4^, ^6^, ^7^ However, certain fetal and maternal factors can have a deleterious effect on the quality of ultrasound image generation, specifically olgohydramnios, advanced gestational age, unfavourable fetal lie, maternal abdominal wall scar, or maternal obesity,^8^ and some elements of diagnosis, such as pulmonary arterial and venous connections, aortic arch morphology, and branching patterns of the head and neck vessels, can be difficult to elucidate using two-dimensional (2D) imaging alone.^4^, ^9^, ^10^, ^11^ Despite major advances in postnatal cardiovascular imaging in the intervening decades, a safe, reliable adjunct to fetal echocardiography has remained elusive. Three-dimensional (3D) fetal echocardiographic techniques, such as spatiotemporal image correlation imaging (STIC), are prone to many of the same limitations as 2D echocardiography, and to fetal motion. Thus these techniques have poor reliability in clinical practice, and have seen limited uptake despite being available for more than 10 years.^12^ MRI, a safe and established adjunct for imaging other fetal organs, such as the fetal brain,^13^ can provide static and dynamic imaging of the fetal heart and vasculature; however, these images are limited to 2D interpretation only, with uncontrolled fetal motion being a major challenge for both acquisition and analysis.^14^, ^15^

**Research in context**

**Evidence before this study**

The antenatal diagnosis of congenital heart disease has become routine in the UK and many other countries with the use of ultrasound, both at 20-week screening scans and at specialist fetal cardiac centres. However, some important aspects of congenital heart disease remain difficult to diagnose with ultrasound, even in the hands of experts, with no reliable alternative. Although several exploratory studies have investigated the potential of two-dimensional (2D) fetal MRI for cardiac imaging, uncontrolled fetal motion during acquisition is consistently cited as a cause of poor reliability, particularly when examining the small, complex three-dimensional (3D) structures of the fetal cardiovascular system. A reliable alternative to ultrasound has thus remained elusive. However, recent innovations in post-hoc image processing algorithms, enabled by advances in modern graphical processing units, have shown promising early results in a research setting, using multiple overlapping 2D MRI images to generate navigable, high-resolution 3D volumes of the brain and thorax in healthy fetuses. This combination has yet to be explored in clinical practice.

**Added value of this study**

By combining standard 2D fetal MRI images with novel motion-corrected slice-volume registration software, we were able to generate a high-resolution, 3D volume of the fetal heart in 85 fetuses with known congenital heart disease. Accuracy of the 3D dataset was validated by comparison with measurements made with paired ultrasound data, with structured clinical analysis showing a significantly better ability to visualise the fetal vascular anatomy compared with source 2D MRI images. In ten fetuses, new anatomical features were described that had previously been undetected, all of which were confirmed postnatally.

**Implications of all the available evidence**

The combination of standard fetal MRI sequences with novel, open-source image processing techniques is a reliable and accurate means of generating detailed 3D imaging of the fetal heart. These methods require minimal additional infrastructure and offer a safe, powerful, and highly complementary adjunct to ultrasound in the diagnosis of congenital heart disease before birth.

However, innovations in post-hoc image processing algorithms have shown promising results in a research setting, with 2D MRI images being used to generate static 3D volumes of the fetal brain,^16^, ^17^ lungs,^18^ and placenta.^19^ Here, we assess the first direct clinical application of these novel computational techniques in the prenatal diagnosis of congenital heart disease, exploring whether they could fulfil a longstanding need for a reliable and accurate form of complementary 3D imaging in this group.

## Methods

### Study design and participants

Pregnant women carrying a fetus with known or suspected congenital heart disease (diagnosed by fetal echocardiography), were recruited via a tertiary fetal cardiology unit, Evelina London Children's Hospital (London, UK). Referrals were encouraged in cases for which 3D visualisation could offer clinically useful complementary imaging, at the discretion of the attending fetal cardiologist (JMS, VZ, GS, OM, TV, or MC). All participants were recruited to one of two long-running fetal imaging studies that allowed for the development of fetal cardiac MRI (REC 07/H0707/105, REC 14/LO/1806) and provided written consent. Eligible women had a pregnancy of 18 weeks or longer at the time of the scan and were aged 18 years or older. Women were excluded if they weighed more than 125 kg, had claustrophobia, had a contraindication to MRI, or were unable to understand study information given in written form and explained verbally.

### Procedures

All fetal data were acquired with a 1·5 Tesla Ingenia MRI system (Philips, Best, Netherlands) using standard T2-weighted single-shot fast spin echo (SSFSE) sequences, which produce black-blood-like contrast between the fetal vessels and the surrounding tissues. Source images consisted of six to 12 multi-slice stacks of 2D images, acquired to cover the fetal thorax in three orthogonal planes. Sequence parameters were: repetition time 20 000 ms, echo time 50 ms, flip angle 90°, voxel size 1·25 × 1·25 mm, slice thickness 2·5 mm, and slice overlap 1·25 mm. Total scan time per stack (approximately 100 slices) was roughly 100 s, depending on patient characteristics. A 3D mask of the fetal thorax was generated from the image stack with the least movement artifact; this stack was also used as the initial registration target. The remaining stacks were then automatically processed using a bespoke motion-correction algorithm, adapted from source code developed by our institutions, and publicly available under a creative commons public licence.^16^, ^17^ In technical terms, the algorithm applies an iterative loop to optimise 2D-to-3D registration on the basis of image intensities, incorporating edge-preserving anisotropic diffusion filtering and automatic exclusion of outlier data. In effect, the software repositions each individual 2D image within a 3D space, aligning them to the correct orientation within an evolving 3D volume, while simultaneously excluding motion-corrupted or noise-corrupted data that vary beyond statistically defined limits. To account for relative or non-rigid transformations between slices (caused, for example, by fetal movement relative to the uterus, fetal limb movement, or maternal respiration) the masked area is treated as a single patch, with computation focused on this region only.^17^ Acceptable computing time (approximately 5–10 min per reconstruction)^17^ was achieved via parallel implementation of the motion compensation algorithms on a graphics processing unit; we used a standard computer with a GeForce GTX TITAN X graphics processing unit.

We did an initial proof-of-concept experiment to assess the general reliability of this technique using data from a 1-day-old neonate and a healthy pregnant volunteer at 38 weeks' gestation (video 1). A standard postnatal 3D balanced steady-state free precession MRI volume was acquired in the neonate under general anaesthetic with cardiac and respiratory gating (the image contrast generated using this sequence is analogous to T2-weighted SSFSE sequences in fetal life, due to the change in lung signal characteristics after birth). The baby had coarctation of the aorta, with a patent arterial duct supported by intravenous prostaglandin, and had cardiac MRI for clinical reasons. No intravenous contrast was used. This volume was then artificially motion-corrupted by simulating nine stacks of 2D images from the 3D dataset and altering the spatial coordinates of each image. To simulate fetal-like motion, nine stacks of 2D MRI images in the same orientation were acquired from the 38-week fetus, and then processed using the reconstruction algorithm according to the methods described above. The spatial coordinates of each image before and after registration were obtained from the log file for the reconstruction; these data were then used to simulate identical displacement in the image stacks simulated from the ground truth neonatal volume. After processing these stacks independently using the motion-correction algorithm, with no a-priori knowledge of the original dataset, we generated a 3D volume of the neonate that was anatomically identical to the original input data (appendix p 1).

To assess the general metric agreement of 3D MRI datasets with ultrasound, when feasible, a paired fetal echocardiogram (Philips EPIQ 7, Philips Healthcare, Bothell, WA, USA), was acquired within 72 h of the MRI by a clinician with experience in fetal cardiology (JMS, TV, or DFAL). These datasets were examined retrospectively by two ultrasound observers (JMS and TV) and two MRI observers (KP and DFAL). Each observer was asked to generate three separate measurements: the mean descending aorta diameter at the level of the left atrium, the mean superior vena cava diameter in a high transverse plane, and the transverse arch diameter in the same plane as the mean superior vena cava diameter (appendix p 2). The echocardiographic observers independently determined the frame of measurement and recorded the widest internal diameter of the vessel of interest. For MRI, the plane of measurement was established by each observer independently navigating a 3D dataset in a multi-planar reconstruction format.

Two observers with expertise in congenital cardiac MRI (HB-R and SM) compared 3D datasets to the source 2D MRI images. A structured anatomical assessment, according to a standard segmental approach, was done consecutively in four areas on the source 2D data, followed by reconstructed 3D data: systemic venous return, pulmonary arterial supply, pulmonary venous return, and aortic and ductal arch anatomy. Two responses were collected for each type of data: first, whether the structure(s) of interest could be visualised or not, and second, if the structure was identified, a diagnostic quality score was given, ranging from 1 (low quality) to 5 (high quality). All observations were made independently and both clinicians were masked to postnatal findings.

All MRI data in this study were contemporaneously reviewed by clinicians with expertise in congenital cardiology (DFAL, KP, RR, and MPMvP, assisted by AS). A full written report was generated for each participant with specific regard to the anatomical areas specified by the referring physician, if applicable; reports were collaborative, with no individual comments, and were uploaded to a secure digital archive. All MRI report findings, along with relevant images, were presented to a multidisciplinary institutional panel before the birth of the affected fetus by one of the same clinicians listed previously. Because the objective was to assess MRI as a secondary diagnostic tool, observers were not masked to echocardiographic findings. If any extra-cardiac fetal abnormalities (new or known) were visualised on MRI, these were reported separately and archived in the same way as cardiac data. After delivery, a definitive cardiac diagnosis was obtained via postnatal imaging or cardiac surgery. Postnatal findings were then systematically compared with the prenatal diagnosis in each case by these same clinicians.

### Statistical analysis

We assessed inter-observer agreement of 3D MRI and ultrasound measurements by calculating the intra-class correlation coefficient, and compared the mean measurements across both methods using the intra-class correlation coefficient. A Kolmogorov–Smirnov test of normal distribution was applied to the mean differences between MRI and ultrasound measurements, after which we did a Bland-Altman anaylsis. We treated mean confidence scores as continuous variables and compared them using a standard *t*-test. When the structure of interest was visualised within both 2D and 3D datasets, we analysed any differences in scoring using a Wilcoxon signed rank test. We did all statistical analysis using SPSS (version 24.0).

### Role of the funding source

The funders had no role in study design, data collection, data analysis, data interpretation, or writing of the report. All named authors had access to all the data in the study and were involved in the decision to submit for publication.

## Results

Between Oct 8, 2015, and June 30, 2017, 101 patients were referred for MRI. All were carrying singleton pregnancies. Three patients fulfilled exclusion criteria: two had claustrophobia and one had undisclosed metal dental braces fitted within 24 h of MRI. Four patients delivered prematurely, before MRI could be performed, and two patients had a medical termination. Two patients could not be scanned because of inpatient medical treatment. A further five patients did not attend for MRI and did not make another appointment, leaving a total of 85 patients. The mean gestational age at MRI was 32 weeks (range 24–36; median 32 weeks [IQR 30–34]). A high-resolution 3D dataset of the fetal thorax (0·50–0·75 mm isotropic) was generated from 2D MRI images in all 85 patients. A full summary of all fetal diagnoses, imaging findings and postnatal diagnosis, is shown in the appendix (pp 5–11).

**Figure 1:**
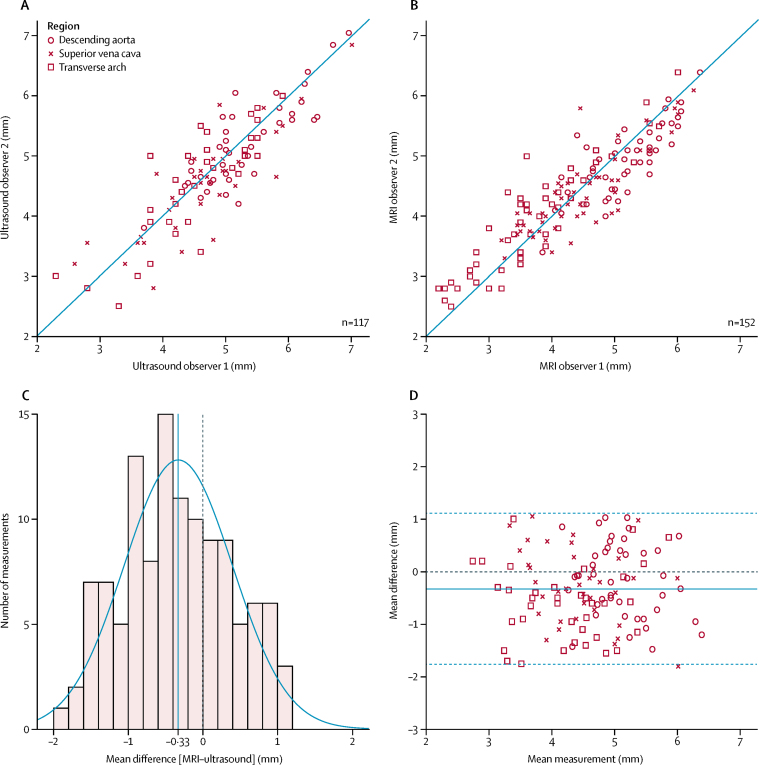
Scatter plots comparing measurements from two independent observers for echocardiography (A) and MRI (B) The blue line represents the line of equality in both panels. The distribution of errors is shown in panel C, with the Bland-Altman plot in panel D. There was a small but significant mean bias of −0·33 mm (95% CI −0·46 mm to −0·20 mm; p<0·0001) in favour of larger echocardiography measurements of the same structure when compared with MRI.

Paired MRI and echocardiography data were available for 51 patients. There was good inter-observer agreement across both methods, with an intraclass correlation coefficient of 0·92 (95% CI 0·89 to 0·95; n=117) for echocardiography and 0·94 (0·92 to 0·96; n=152) for MRI (figure 1A, 1B). There was moderate agreement between the mean measurements of the same structure across both methods (n=117), with an intraclass correlation coefficient of 0·78 (95% CI 0·68 to 0·84). The mean difference between MRI and echocardiographic measurements was −0·33 mm (95% CI −0·46 mm to −0·20 mm; p<0·0001), suggesting a small but significant systematic bias towards smaller values from MRI compared with 2D echocardiographic measurements. A Kolmogorov–Smirnov test done on the mean differences resulted in a p value of 0·20, suggesting a normal distribution of errors (figure 1C); a Bland-Altman plot of these measurements is shown in figure 1D.

**Figure 2:**
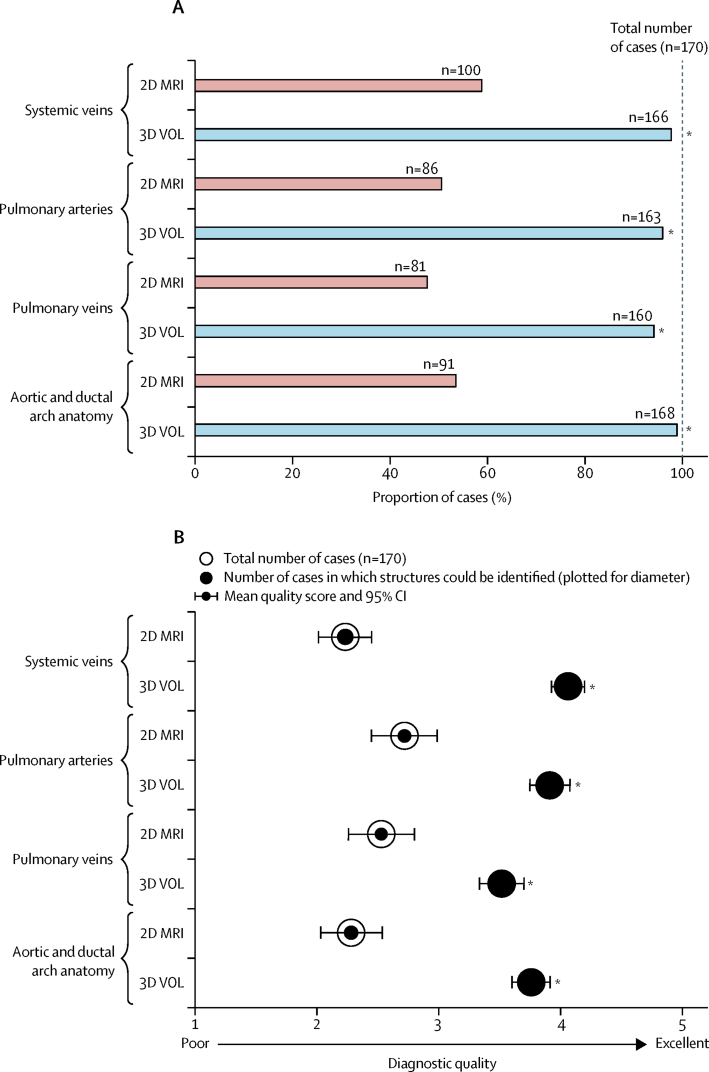
Results of structured anatomical assessment from two independent observers, shown by anatomical category (A) Proportion of all cases in which structures of interest could be identified. (B) Diagnostic quality assessment. 2DMRI=two-dimensional MRI data. 3DVOL=three-dimensional motion-corrected MRI volume. *p<0·0001.

We did a structured assessment of 2D and 3D MRI data in all 85 patients. Each observer was asked to identify four anatomical areas in each fetus; these were identified in 358 (53%) of 680 instances in the 2D datasets, and 657 (97%) out of 680 instances in 3D data (p<0·0001). Figure 2A shows these results across the four major anatomical categories. When the structure of interest was identified, the mean overall quality score for 2D data was 2·4 out of 5 (median 2, IQR 1–3; n=358), increasing to 3·8 for 3D data (median 4, IQR 3–5; n=657; p<0·0001). When the structure of interest was visualised in both 2D and 3D data (n=358), observers gave a higher score for 3D data in 321 (90%) of cases, with 37 (10%) scores tied with 2D data, and no lower scores than for 2D data (Wilcoxon signed rank test p<0·0001). Mean scores analysed by anatomical category are shown in figure 2B.

**Figure 3:**
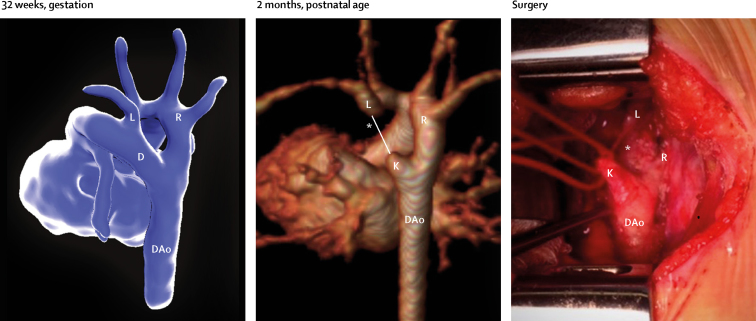
Motion-corrected MRI data from a fetus with double aortic arch at 32 weeks Shown are the descending aorta (DAo), arterial duct (D), and left (L) and right (R) aortic arches. At 2 months postnatal age, contrast-enhanced MRI could show a right-sided arch (middle panel); however, a ligamentous remnant of the left arch was predicted on the basis of the fetal MRI findings (asterisk); this finding was confirmed at surgery (right panel). The distal remnant of the arterial duct—analogous to the diverticulum of Kommerell—is also seen (K). See video 3 for more detail.

**Figure 4:**
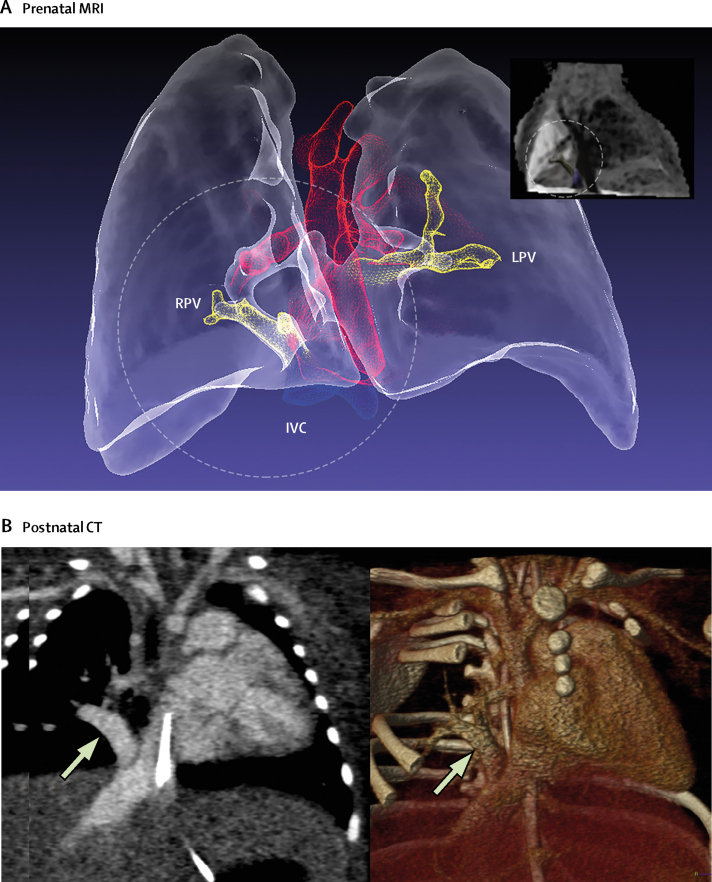
Example segmentation from motion-corrected 3D data, in a 33-week fetus referred because of abnormal right pulmonary veins on echocardiography (A) In this anterior projection of the lungs and major blood vessels, a single right pulmonary vein (RPV) can be seen draining anomalously to the inferior vena cava (IVC) in the circled area. A single left pulmonary vein (LPV) was also noted. Inset: minimum-intensity projection of the 0·7mm isotropic volume used to generate segmentation (RPV=yellow, IVC=blue). (B) All findings were confirmed postnatally by contrast-enhanced CT, with the anomalous vein indicated with an arrow. See video 4 for more detail.

**Figure 5:**
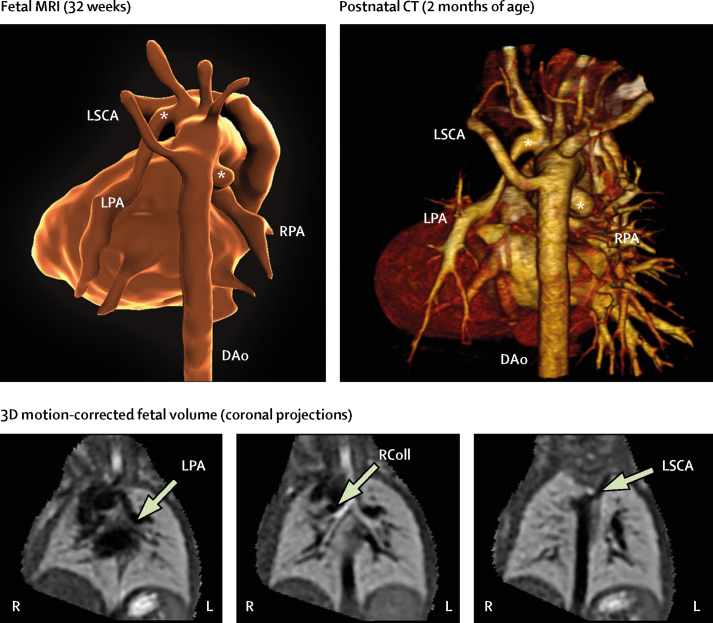
Segmentation of a fetal heart from 3D data at 32 weeks in a fetus with pulmonary atresia and ventricular septal defect, right aortic arch, and disconnected pulmonary arteries Fetal MRI showed two large collateral arteries (asterisks): the left pulmonary artery (LPA) was supplied by a large collateral vessel originating close to the origin of the left common carotid artery, with the right pulmonary artery (RPA) supplied by a tortuous collateral or arterial duct from the underside of the aortic arch. The left subclavian artery (LSCA) was also noted to be aberrant. All findings were confirmed with postnatal CT at 2 months (right panel). The bottom panel shows three coronal planes of the original 3D motion-corrected fetal data. These images were used for pre-birth surgical planning and parental counselling. DAo=descending aorta. RColl=right collateral.

A prenatal MRI report was generated for all patients, which was based on the analysis of both 2D and motion-corrected 3D data. Reports were focused on the aspects of fetal anatomy specified by the referring consultant (appendix pp 5–11), and each report was completed, presented, and archived before birth of the fetus. Example images from 3D reconstructed data are shown in Figure 3, Figure 4, Figure 5; appendix p 3; videos 2–4). Definitive postnatal diagnosis was available in 83 cases, via cardiac surgery (n=35), echocardiography (n=32), CT (n=12), MRI (n=2), or cardiac catheterisation (n=2). Two babies died soon after birth without definitive postnatal imaging: one baby with a large pulmonary arteriovenous malformation, and one with hypoplastic left heart syndrome and total anomalous pulmonary venous drainage. In both cases, a decision was made with the family to proceed with compassionate care only. No post-mortems were done.

Analysis of MRI detected additional anatomical findings in ten cases (four cases with right aortic arch found to have an aberrant left subclavian artery, three cases found to have a retro-aortic innominate vein, two cases with right aortic arch found to have a double aortic arch, and one case found to have bilateral superior vena cavas; all confirmed postnatally). One case with suspected coarctation was found postnatally to have an anomalous left upper pulmonary vein, draining to the innominate vein, which had not been reported on fetal ultrasound or MRI. A summary of the diagnostic pathway for all patients referred is shown in the appendix (p 4).

**Figure 6:**
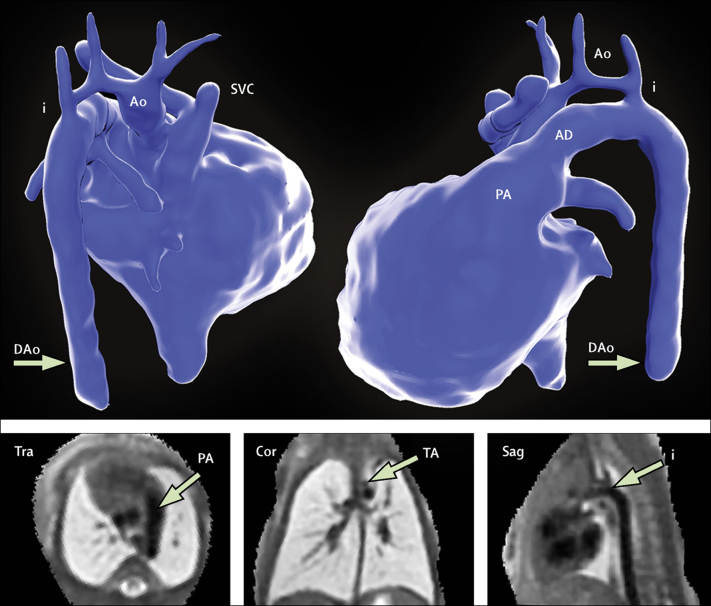
Segmentation of motion-corrected MRI data of a fetus with suspected coarctation of the aorta at 33 weeks' gestation Posterior projection (top left) and left lateral projection (top right) are shown. The aorta (Ao), arterial duct (AD), descending aorta (DAo), aortic isthmus (i), and superior vena cava (SVC) are labelled. Coarctation was confirmed after birth and treated surgically. The bottom panel shows planes from the reconstructed 3D dataset in a transverse (Tra), coronal (Cor), and sagittal (Sag) orientation. See video 2 for more detail. TA=transverse arch. PA=pulmonary artery.

Three predominant diagnostic categories emerged from the referral pattern for additional imaging. The first was aortic arch hypoplasia and suspected coarctation of the aorta (n=34). The difficulties in accurately predicting postnatal coarctation of the aorta with prenatal imaging are well described.^20^ Prognostic features based on the geometry of the aortic arch with respect to the arterial duct have been described using 2D echocardiography; however, specificity with these techniques is poor.^21^, ^22^ 3D analysis allows for a more detailed depiction of the aortic arch morphology (figure 6; video 2). Analysis of these data, in combination with other advanced echocardiographic and MRI techniques,^23^ is the focus on ongoing research within our departments.

The second diagnostic category was right-sided or double aortic arch (n=27). The incidence of isolated right or double aortic arch has increased markedly since the incorporation of the high mediastinal view into routine screening.^24^, ^25^ When there is an aberrant origin of the left subclavian artery or double aortic arch, important tracheoesophageal compression can become apparent in early postnatal life, requiring surgical intervention. Double aortic arch can be difficult to visualise before birth,^24^ and indeed after birth in cases when the distal left arch becomes atretic following closure of the arterial duct (a phenomenon that might be under-recognised).^25^ 3D fetal imaging might directly inform postnatal imaging and further management in these cases (figure 4; video 3). Concomitant fetal airway imaging with 2D MRI could help stratify postnatal risk further.

The final diagnostic category was pulmonary vasculature (n=15). Abnormalities of the pulmonary arteries and veins have the greatest overall effect on long-term treatment when they are identified before birth.^4^ They are also among the most difficult abnormalities to diagnose. Aortopulmonary collaterals, for example, can take myriad forms, with 2D echocardiographic assessment often complicated by multiple vessels passing in and out of a single sonographic plane.^9^, ^11^ Anomalous pulmonary venous drainage can be similarly difficult to define.^10^ 3D visualisation can be a powerful adjunct in these cases (Figure 5, Figure 6; video 4). Concomitant 2D MRI imaging of the fetal lungs can help to evaluate for evidence of pulmonary lymphangiectasia, an important determinant of long-term outcome in pulmonary venous obstruction.^26^ One fetus in this study, with hypoplastic left heart syndrome and obstructed pulmonary venous return, had features of lymphangiectasia. The baby died at one day of age.

## Discussion

2D echocardiography is the mainstay for the diagnosis of congenital heart disease before birth. It is a safe and highly sensitive bedside test that can be employed from the first trimester of pregnancy.^6^ However, there is an established and longstanding desire for a reliable adjunct to ultrasound, either because of patient-specific limitations, or in certain forms of congenital heart disease, when 3D visualisation could offer complementary anatomical information. In this study, we assessed the combination of fetal MRI with advanced open-source image processing algorithms, in terms of reliability, veracity, and clinical demand, in 85 fetuses with a range of congenital heart defects. To our knowledge, this is the largest study to date exploring the use of prenatal MRI in fetuses with congenital heart disease.

The effectiveness and reliability of most 2D MRI techniques is heavily influenced by fetal motion.^8^, ^15^ This limitation can lead to high incidence of failure in identifying the structure of interest,^15^ with the loss of anatomical continuity between adjacent slices often impeding more detailed interrogation (video 1). By combining standard 2D MRI images with the motion-correction algorithms described in this study, we were able to generate a high-resolution, 3D output in all fetuses, with 90% of reconstructions showing improved diagnostic quality over the source data. In ten patients, these datasets allowed for the identification of new anatomical features that had not been previously described. All imaging in this study was generated from standard MRI sequences with widely available, non-modified computer hardware and software. We would anticipate less than 30 min of total MRI time and 30 min of post-processing to be ample for the majority of patients using our systems.

The ability to provide comprehensive and accurate prenatal confirmation of cardiovascular anatomy is potentially very valuable, particularly for conditions in which echocardiography can be limited, such as abnormalities of the pulmonary blood supply.^9^, ^10^ These patients might otherwise have waited until postnatal imaging was done to attain a similar level of confidence in diagnosis. The addition of 2D MRI imaging of the fetal lungs, airways, or other organ systems can add further valuable prognostic information in selected cases.^26^ A comprehensive fetal diagnosis allows for unambiguous fetal counselling, as well as definitive planning of early postnatal management and surgical approach, well before the fetus is exposed to the risks of the transitional circulation.

Although these image processing techniques have previously been validated in a research setting,^16^ this is their first application in clinical practice. The veracity of the 3D datasets was therefore explored in two ways: first, by showing anatomical congruity between a ground truth neonatal MRI volume and reconstructed 3D data from simulated fetal motion (appendix p 1) and second, in the correlation between MRI and ultrasound measurements in 51 cases with paired imaging data (figure 1). Both results were reassuring. The small but significant bias in favour of larger echocardiographic measurements could be explained by the fact that these measurements were taken at their widest point in the cardiac cycle, whereas non-gated 3D MRI volumes are effectively static averages of the maximum and minimum vascular dimensions.

Spatiotemporal image correlation is the most well described and widely available ultrasound method for 3D visualisation of the fetal vasculature. The modern version of spatiotemporal image correlation utilises a virtual 2D sweep of images from a 3D matrix array ultrasound probe, re-ordered into a single cardiac cycle to produce a four-dimensional volume for offline interrogation. The intra-cardiac anatomy is also represented, and the technique has been described as early as the first trimester. Despite these potential advantages, the technique is hindered by poor reliability in practice, with failure to obtain suitable volumes for further analysis in a third to two-thirds of cases.^12^, ^27^ The reliance on imaging from a single ultrasound probe means this method shares many similar limitations to general ultrasound, and, although the process only lasts a few seconds, even small amounts of fetal or maternal motion can generate substantial stitching artifacts. The final volume is also non-isotropic, with reduced resolution in the imaging plane perpendicular to the usual acquisition planes of the probe. The MRI technique described in this study might be a promising and reliable alternative in cases where ultrasound assessment is limited. Looking forward, prospective studies directly comparing MRI with echocardiography could help to identify a robust set of fetal indications for further imaging, similar to those developed in postnatal life.

The software-based registration technique we describe is one thread amongst several exploring the potential marriage of fetal MRI with novel computational techniques. For example, methods to combine single-slice motion-correction with temporal re-ordering have been used to generate high-resolution 2D cine loops of fetal heart motion.^14^ Combining these methods with those described here could allow for the generation of a fully spatiotemporally resolved, high-resolution four-dimensional volume of the fetal heart from MRI.^28^ Post-hoc cardiac gating methods such as metric-optimised gating^29^ have been used with phase contrast sequences to measure vascular flow rates in several scenarios, with T1 and T2 mapping techniques being investigated to estimate intravascular oxygen saturations.^30^ The combination of these techniques could allow for a sophisticated multi-parametric analysis of the fetal cardiovascular system, akin to that available in postnatal life. Alongside advanced fetal ultrasound techniques^23^ these tools could provide important insights in difficult prenatal diagnoses, such as coarctation of the aorta, as well as other strongly linked pathology, such as abnormal brain development,^17^, ^31^ placental dysfunction,^19^ and long-term cardiovascular health.

The timing for MRI in this series was chosen prospectively because we felt it provided the optimum balance in terms of fetal size, stillness, and resolution. Most studies were therefore done in the early third trimester. The smaller size and increased potential range of movement of younger fetuses might affect the reliability of this technique at earlier gestation and was not formally investigated; this is an important area on which to focus future modifications. For safety and comfort in the MRI scanner, we excluded patients who weighed more than 125 kg. However, these patients can be difficult to image with ultrasound, and thus could be an important group to benefit from alternative imaging. The relative decrease in signal-to-noise ratios in heavier patients might be offset by the use of overlapping data and removal of outlying voxels in the registration algorithm. This is also an important area for future research.

Fetal cardiovascular MRI, incorporating novel open-source 3D image processing algorithms, can significantly improve the visualisation of major vascular abnormalities in late-gestation fetuses compared with 2D MRI. This freely available combination requires minimal additional infrastructure, and offers the potential for a safe, reliable and highly complementary form of imaging of the fetal cardiovascular system.

## Supplementary Materials

Supplementary video 1 An example of the three-dimensional reconstruction pipeline. A stack of two-dimensional MRI images is shown in the acquired plane; movement artefact between slices prevents interpretation when the stack is viewed from any other direction. After motion correction, the vessels of the thorax can now be fully explored in three dimensions. This can then be used to produce a detailed three-dimensional model of the heart, shown here matched to standard echocardiographic planes.

Supplementary video 2 The aortic arch and isthmus are highlighted on prenatal echocardiography in a fetus with suspected coarctation of the aorta, a condition known to be difficult to detect before birth. Motion-corrected three-dimensional MRI at 32 weeks provides a detailed three-dimensional representation of the size, position and relationship of these vessels. Coarctation of the aorta was confirmed after delivery.

Supplementary video 3 Motion-corrected MRI data in a fetus at 32 weeks' gestation shows a double aortic arch with dominant right arch (R), smaller left arch (L) and left sided arterial duct (D) producing a vascular ring encircling the trachea. The left arch was not seen on contrast-enhanced MRI at 2 months postnatal age (middle panel); however, a ligamentous remnant of this was predicted on the basis of the fetal MRI findings (asterisk). This was confirmed at surgery (right panel). The distal remnant of the arterial duct (K) is see connected to the descending aorta (Ao).

Supplementary video 4 The right-sided pulmonary veins are shown on two-dimensional and colour Doppler echocardiography at 24 and 31 weeks' gestation, with no clear connection to the left atrium. Three-dimensional MRI data acquired at 33 weeks' gestation confirmed the right pulmonary veins to be draining abnormally to the inferior vena cava, shown here in a minimum intensity projection, and as a three-dimensional model of the heart and lungs viewed from a posterior aspect. Pulmonary veins are yellow. Systemic veins are blue.
