# Department of Error

**DOI:** 10.1016/S0140-6736(22)00702-4

**Published:** 2022-04-23

*Lloyd DFA, Pushparajah K, Simpson JM, et al. Three-dimensional visualisation of the fetal heart using prenatal MRI with motion-corrected slice-volume registration: a prospective, single-centre cohort study.* Lancet *2019; **393:** 1619–27*—In this Article, Alexander Schulz's affiliations should have included Charité – Universitätsmedizin Berlin, corporate member of Freie Universität Berlin and Humboldt-Universität zu Berlin, Berlin, Germany. This correction has been made to the online version as of April 21, 2022.

