
JOURNAL INFORMATION
==============================
NLM Title Abbreviation: Lancet
Journal ID: Lancet
NLM Journal ID: 2985213R

Lancet (London, England)

ISSN: 0140-6736
EISSN: 1474-547X

ARTICLE INFORMATION
==============================
PMCID: PMC9152948
PMID: 35598623
DOI: 10.1016/S0140-6736(22)00913-8
Article ID: S0140-6736(22)00913-8
Article version: 1
Subjects: Department of Error

Department of Error

Print publication date: 2022 May 21
Volume: 399
Issue: 10339
First page: 1940
Last page: 1940
Copyright: © 2022 The Author(s). Published by Elsevier Ltd.
Copyright year: 2022
Copyright holder: Elsevier Ltd
License: This is an open access article under the CC BY license (http://creativecommons.org/licenses/by/4.0/).
License URL: https://creativecommons.org/licenses/by/4.0/

Erratum for: Three-dimensional visualisation of the fetal heart using prenatal MRI with motion-corrected slice-volume registration: a prospective, single-centre cohort study 393 10181 20 4 2019 1619 1627 Lancet (London, England) 10.1016/S0140-6736(18)32490-5 PMC6484696 30910324

==============================
Lloyd DFA, Pushparajah K, Simpson JM, et al. Three-dimensional visualisation of the fetal heart using prenatal MRI with motion-corrected slice-volume registration: a prospective, single-centre cohort study. Lancet 2019; 393: 1619–27—In this Article, Alexander Schulz's affiliations should have included Charité – Universitätsmedizin Berlin, corporate member of Freie Universität Berlin and Humboldt-Universität zu Berlin, Berlin, Germany. This correction has been made to the online version as of May 19, 2022.

