# Supplementary material for: Three-dimensional visualisation of the fetal heart using prenatal MRI with motion-corrected slice-volume registration: a prospective, single-centre cohort study
Source: Lancet. 2019 Apr 20;393(10181):1619–27. doi: 10.1016/S0140-6736(18)32490-5 (PMC6484696; doi:10.1016/S0140-6736(18)32490-5)
Supplement: Supplementary appendix [file mmc5.pdf]

THE LANCET

Supplementary appendix

This appendix formed part of the original submission and has been peer reviewed. We post it as supplied by the authors.

Supplement to: Lloyd DFA, Pushparajah K, Simpson JM, et al. Three-dimensional visualisation of the fetal heart using prenatal MRI with motion-corrected slice-volume registration: a prospective, single-centre cohort study. *Lancet* 2019; published online March 22. http://dx.doi.org/10.1016/S0140-6736(18)32490-5.

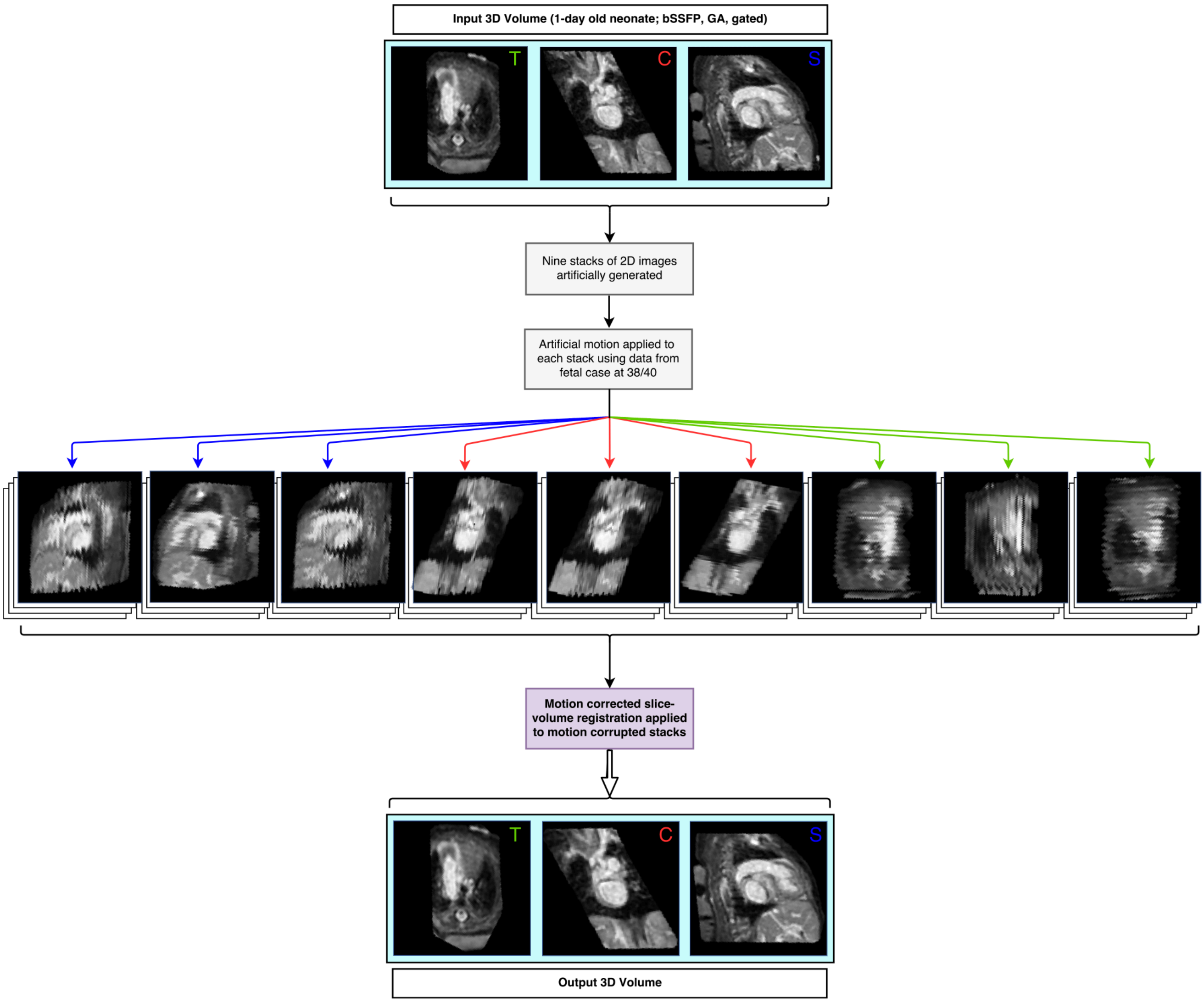

Supplementary Figure 1. Summary of initial validation experiment. Three orthogonal planes from a cardiac and respiratory gated three-dimensional balanced steady-state free precession (bSSFP) MRI volume acquired in a one-day old baby under general anaesthetic (GA) (top panels), displayed in a transverse (T), coronal (C) and sagittal (S) orientation. This volume was used to synthesise nine motion corrupted "stacks" of single slices, equivalent to the data obtained using standard 2D MRI sequences in a moving fetus (middle panels). The motion-correction algorithm described in this report was then applied, using only these stacks as input data. The new 3D volume generated was anatomically identical to the original 3D bSSFP MRI (bottom panel).

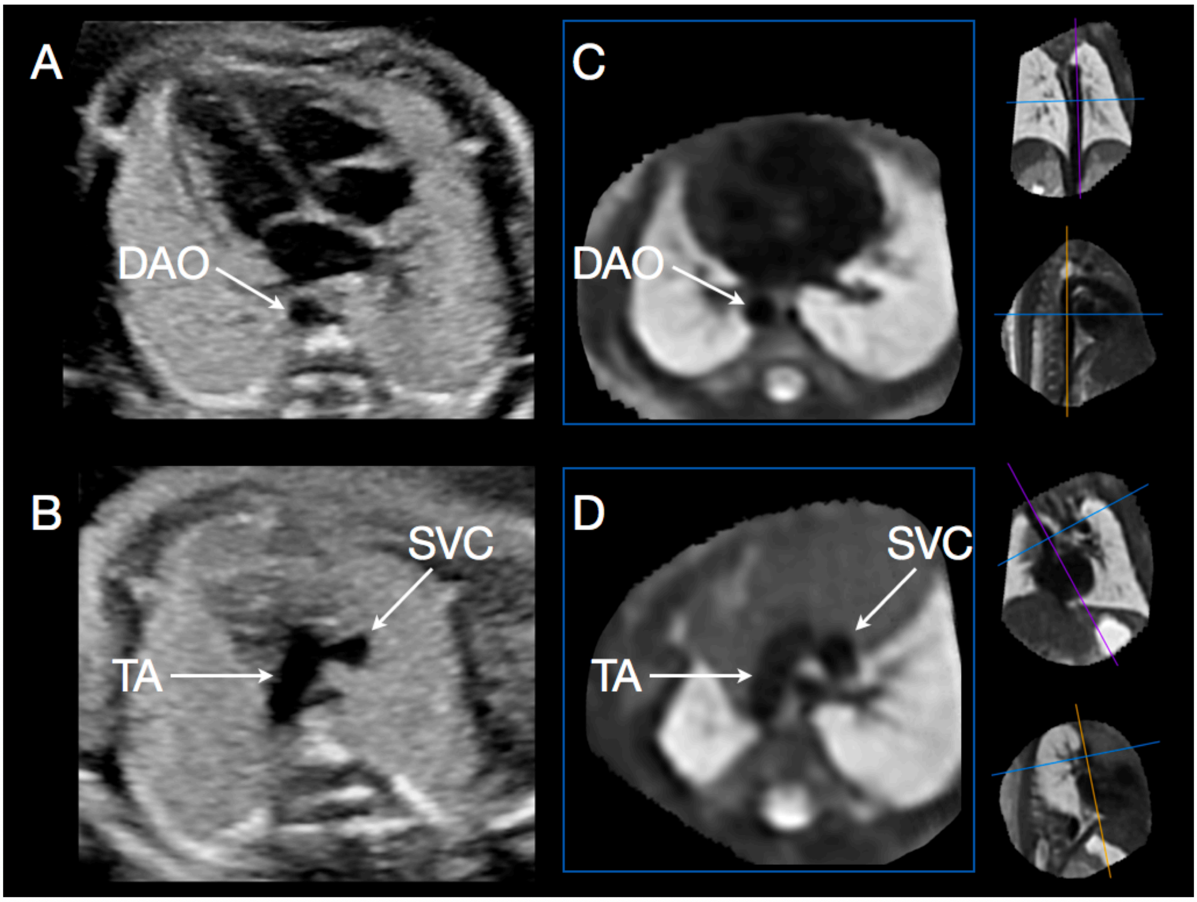

Supplementary Figure 2. Summary of retrospective measurements taken by two echocardiographic and two MRI observers in fetuses with paired US/MRI datasets. The mean of two cross sectional diameters of the descending aorta (DAO) was measured in the echocardiographic four chamber view (panel A), with the mean superior vena cava (SVC) diameter and single transverse arch (TA) diameter measured in a high transverse "three vessel trachea" view (panel B). The same structures were measured separately by two independent MRI observers (panels C & D), using multi-plane reconstruction software to navigate a three-dimensional MRI volume.

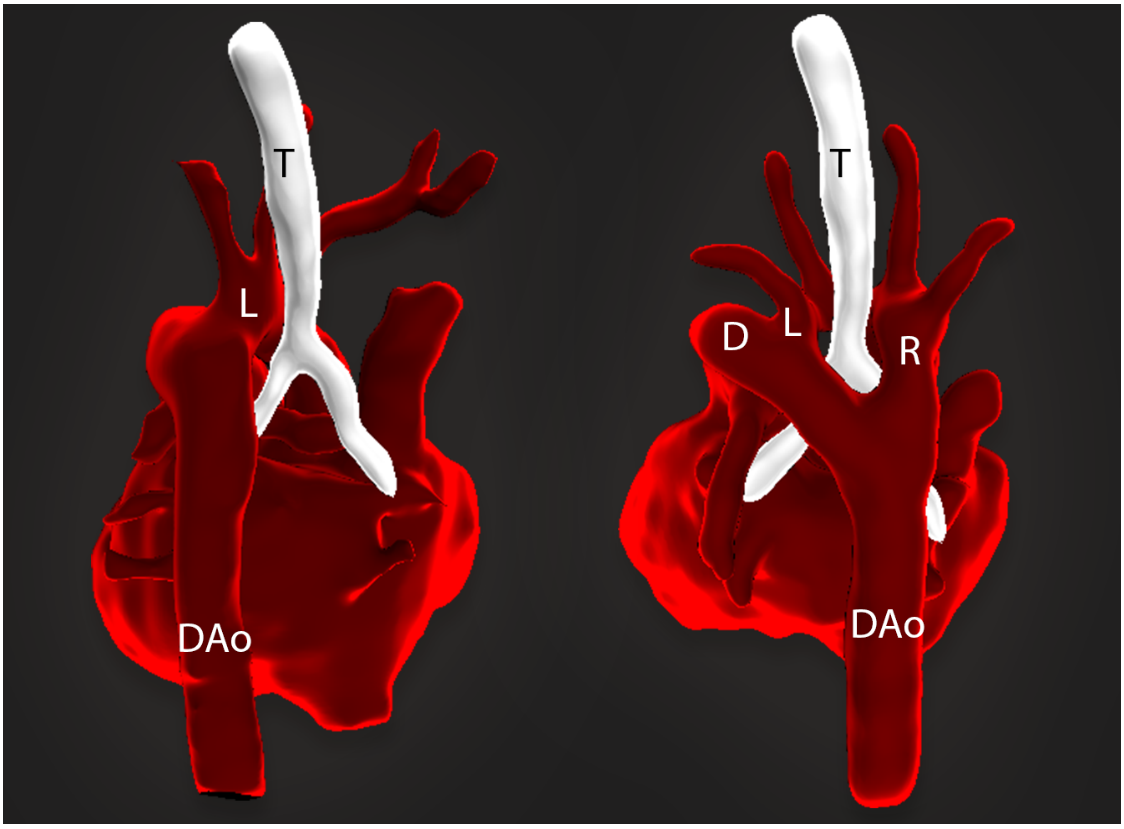

Supplementary Figure 3. Posterior view of a normal fetal heart at 38 weeks (left) and double aortic arch at 32 weeks (right), showing the descending aorta (DAo), arterial duct (D), and left (L) and right (R) aortic arches. Both images have been segmented from motion-corrected fetal MRI data. The trachea (T) is also shown, demonstrating how the latter produces a "vascular ring" encompassing the mediastinum.

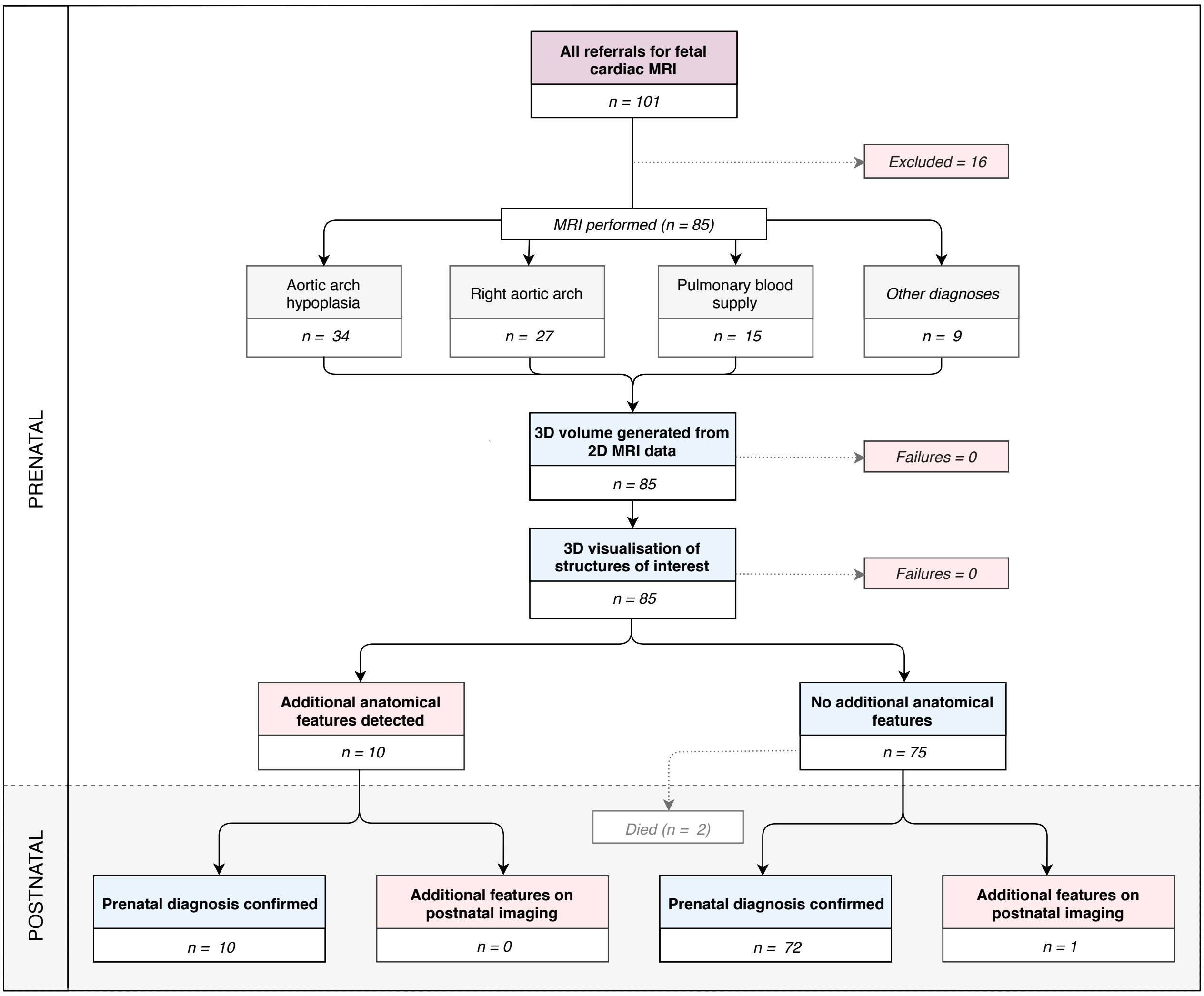

Supplementary Figure 4. Flowchart showing the pathway for all patients referred for prenatal cardiac MRI.

Supplementary table: summary of all patients undergoing fetal cardiac MRI

N	GA Echo	Echocardiographic diagnosis	MRI area of interest	GA MRI	MRI findings	MRI additional information	Postnatal diagnosis (PND)	Modality	Age
**1**	28	• Hypoplastic left heart syndrome<br>• Mitral and aortic atresia<br>• Restrictive atrial septum<br>• Non-dilated left atrium	*Pulmonary veins: possibility of decompressing vein*	31	• **Normal pulmonary venous drainage**<br>• **No decompressing vein**<br>• Left aortic arch with normal branching pattern	• No evidence of pulmonary lymphangectasia	• Hypoplastic left heart syndrome<br>• Normal pulmonary venous drainage	Surgery	3d
**2**	23	• Hypoplastic right lung<br>• Cardiac dextroposition<br>• Cannot exclude Scimitar syndrome	*Right sided pulmonary venous anatomy*	24	• **No visible right lung, right pulmonary veins, right pulmonary arteries**	• Normal arterial and venous supply to the left lung<br>• No arterial or venous collaterals visualised	• Agenesis of right lung	CT	14d
**3**	26	• VSD with overriding aorta<br>• Likely common arterial trunk<br>• Unable to visualise branch pulmonary arteries<br>• Cannot exclude pulmonary atresia with ventricular septal defect	*Pulmonary arterial blood supply*	31	• **Two large collaterals from anterior descending aorta**<br>• **No native branch pulmonary arteries**<br>• Right aortic arch with mirror image branching	• No visible thymus tissue	• Pulmonary atresia with ventricular septal defect<br>• Major aortopulmonary collateral arteries<br>• No native pulmonary arteries<br>• 22q11 microdeletion syndrome	Catheter	11d
**4**	28	• Transposition of the great arteries<br>• Ventricular septal defect<br>• Likely coarctation of the aorta	-	34	• Narrow distal aorta, tapering significantly towards isthmus	-	• Transposition of the great arteries<br>• Distal aortic hypoplasia of arch, tapering at transverse arch to isthmus<br>• Coarctation of the aorta	Surgery	19d
**5**	31	• Balanced four chamber view<br>• Muscular ventricular septal defect<br>• Slender transverse arch<br>• Possible coarctation of the aorta	*?Evidence of coarctation of the aorta*	36	• **Elongated and mildly hypoplastic aortic arch**<br>• Normal aortic arch branching pattern	-	• Slender arch with borderline hypoplasia<br>• No coarctation or the aorta	Echo	414d
**6**	31	• Right aortic arch<br>• Left arterial duct<br>• Possible double aortic arch	*Rue out double aortic arch*	32	• **Double aortic arch**<br>• **Dominant right and smaller left sided aortic arch**	• No evidence of segmental lung pathology<br>• Mild compression right trachea	• Double aortic arch<br>• Hypoplasia/coarctation of left arch<br>• 90% compression distal trachea (bronchoscopy)	Surgery	80d
**7**	31	• Pulmonary atresia, ventricular septal defect<br>• Aortopulmonary collateral arteries<br>• Possible small central pulmonary arteries<br>• Aberrant right subclavian artery	*Pulmonary arterial blood supply*	31	• **Large aortopulmonary collaterals from anterior descending aorta**<br>• **No native branch pulmonary arteries**<br>• **No arterial duct**	• No visible thymus tissue	• Pulmonary atresia with ventricular septal defect<br>• Major aortopulmonary collateral arteries from anterior descending aorta<br>• No native branch pulmonary arteries	Catheter	23d
**8**	32	• Common arterial trunk<br>• Small aortic arch<br>• Possible coarctation of the aorta<br>• Normal origin of right subclavian artery	*?Evidence of coarctation of the aorta*	32	• **Aortic arch hypoplasia with significant isthmal narrowing**<br>• Separate origins of pulmonary arteries from common arterial trunk	• Normal thymus	• Common arterial trunk (type 2)<br>• Hypoplastic aortic arch<br>• Severe coarctation of the aorta	Surgery	8d
**9**	32	• Aneurysmal atrial septum<br>• Ventricular disproportion<br>• Slender aortic arch<br>• Possible coarctation of the aorta	*?Evidence of coarctation of the aorta*	35	• **Mild distal arch hypoplasia only**	-	• Mild distal arch hypoplasia<br>• No coarctation postnatally	Echo	408d
**10**	34	• Atrial flutter (resolved)<br>• Atrial septal aneurysm<br>• Retrograde flow in the aortic arch<br>• Possible coarctation of the aorta	*?Evidence of coarctation of the aorta*	35	• **Normal calibre aortic arch and isthmus**	-	• Normal arch dimensions<br>• No coarctation postnatally	Echo	185d
**11**	33	• Ventricular and great artery disproportion<br>• Tapering of the distal transverse aortic arch<br>• High suspicion of coarctation of the aorta	-	33	• Distal tapering of arch with narrow aortic isthmus	-	• Mild hypoplasia of distal aortic arch and isthmus<br>• No coarctation postnatally	Echo	76d

**12**	34	• Mitral atresia<br>• Hypoplastic ascending aorta<br>• Possible interruption of the aortic arch	*?Interruption of the aortic arch*	34	• **Severe aortic arch hypoplasia (not interrupted)**<br>• Great artery disproportion	-	• Severe arch hypoplasia with coarctation<br>• CHARGE association	Echo	3d
**13**	31	• Levocardia with left axis deviation<br>• Ventricular asymmetry<br>• Left sided pulmonary veins to left atrium<br>• Right sided pulmonary veins not clearly demonstrated<br>• Branch pulmonary arteries not seen	*Pulmonary venous drainage*<br>*Pulmonary arterial supply*	33	• **Partial anomalous pulmonary venous drainage (right side pulmonary veins to inferior vena cava)**<br>• **Single left sided pulmonary vein**<br>• **Normal branch pulmonary artery origins**	-	• Partial anomalous pulmonary venous drainage (right side pulmonary veins to inferior vena cava)<br>• Confluent branch pulmonary arteries	CT	27d
**14**	28	• Left heart hypoplasia<br>• Aortic arch hypoplasia<br>• Muscular ventricular septal defect<br>• Possible aberrant right subclavian artery	*Confirm arch morphology/ branching pattern*	30	• **Left aortic arch with aberrant right subclavian artery**<br>• Generalised hypoplasia of the ascending aorta and aortic arch	-	• Left aortic arch with aberrant right subclavian artery<br>• Hypoplasia of the ascending aorta and aortic arch	MRI	92d
**15**	35	• Tetralogy of Fallot with absent pulmonary valve<br>• Right aortic arch<br>• Ductus arteriosus not seen<br>• Retro aortic innominate vein	-	33	• Grossly dilated pulmonary arteries<br>• Absent arterial duct<br>• Retroaortic innominate vein	• Thymus gland present<br>• No evidence of segmental lung pathology	• Tetralogy of Fallot with absent pulmonary valve<br>• Dilated pulmonary arteries<br>• Significant airway compression<br>• Normal genetics	MRI	99d
**16**	28	• Ventricular and great artery disproportion<br>• Possible dysplastic mitral valve<br>• Hypoplastic aortic arch<br>• Possible coarctation of the aorta	*?Evidence of coarctation of the aorta*	33	• **Generalised aortic arch and isthmal hypoplasia**	-	• Borderline left heart<br>• Generalised aortic arch hypoplasia<br>• Coarctation of the aorta	Surgery	10d
**17**	33	• Transposition of the great arteries<br>• Outlet ventricular septal defect<br>• Great artery disproportion<br>• Likely coarctation of the aorta	-	35	• Distal arch tapers significantly distal to the left subclavian	-	• Severe coarctation with small strand of tissue distal to left subclavian	Surgery	5d
**18**	31	• Mitral atresia<br>• Double outlet right ventricle<br>• Severe aortic arch hypoplasia<br>• ?Abnormal drainage of pulmonary veins	*Confirm pulmonary venous anatomy*	31	• **Supracardiac total anomalous pulmonary venous drainage with potential obstruction to ascending vein between the ductal arch and left pulmonary artery**<br>• Generalised aortic hypoplasia	-	• Obstructed supracardiac total anomalous pulmonary venous drainage<br>• Coarctation of the aorta	Echo	1 day
**19**	23	• Right sided aortic arch<br>• Possible ventricular septal defect<br>• Aberrant left subclavian artery	-	29	• Right aortic arch with aberrant left subclavian artery<br>• Left sided arterial duct	• Mild distortion of trachea noted	• Right aortic arch with aberrant left subclavian artery<br>• Right sided tracheal compression (bronchoscopy)	CT	37d
**20**	33	• Tetralogy of Fallot with absent pulmonary valve<br>• Dilated right and left pulmonary arteries<br>• No arterial duct seen	-	33	• Grossly dilated pulmonary arteries<br>• Absent arterial duct	• Thymus gland present<br>• No evidence of segmental lung pathology	• Tetralogy of Fallot with absent pulmonary valve<br>• Grossly dilated branch pulmonary arteries<br>• Absent arterial duct<br>• No airway compression (bronchoscopy)	CT	112d
**21**	27	• Atrioventricular septal defect<br>• Double outlet right ventricle<br>• Pulmonary atresia<br>• Major aortopulmonary collateral arteries<br>• Possible hypoplastic native pulmonary arteries	*Pulmonary blood supply*	30	• **Confluent but hypoplastic pulmonary arteries**<br>• **Major aortopulmonary collateral arteries visualised arising from descending aorta**<br>• **Bilateral superior vena cavas, no bridging vein**	• Thymus gland present	• Tetralogy of Fallot<br>• Confluent but hypoplastic pulmonary arteries.<br>• Major aortopulmonary collateral arteries visualised arising from descending aorta<br>• Bilateral superior vena cavas, no bridging vein<br>• Normal genetics	CT	10d
**22**	31	• Apex of the heart to the right<br>• Ventricular septal defect<br>• Possible sub aortic stenosis<br>• Hypoplastic aortic arch<br>• Likely coarctation of the aorta	-	33	• Significant aortic arch hypoplasia, particularly transverse portion	• No evidence heterotaxy syndrome	• Transverse arch hypoplasia<br>• Coarctation of the aorta	Surgery	15d
**23**	27	• Ventricular and great artery disproportion<br>• Hypoplastic aortic arch<br>• Likely coarctation of the aorta	-	30	• Transverse arch hypoplasia with isthmus inserting onto ductal arch	-	• Significant hypoplasia of the aortic arch distal the left common carotid artery<br>• Coarctation of the aorta	Surgery	3d

**24**	21	• Isolated right aortic arch<br>• Left arterial duct<br>• Smaller left sided arch cannot be ruled out	*Rule out double aortic arch with hypoplastic left arch*	31	• **Right aortic arch with aberrant left subclavian artery and left sided arterial duct**	• No segmental lung pathology/evidence of tracheal obstruction	• Right aortic arch with aberrant left subclavian artery and left sided arterial duct	Surgery	324d
**25**	26	• Transposition of the great arteries<br>• Mid-muscular ventricular septal defect<br>• Suspicion of coarctation of the aorta	*?Evidence of coarctation of the aorta*	29	• **Transposed great vessels**<br>• **Left aortic arch with normal branching**<br>• **Arch tapers distal to left subclavian to diameter of <2mm at the isthmus**	-	• Transposition of the great arteries<br>• Mid-muscular ventricular septal defect<br>• Coarctation of the aorta	Surgery	14d
**26**	35	• Ventricular septal defect<br>• Likely coarctation of the aorta	-	36	• Left sided aortic arch with common origin of brachiocephalic and left common carotid<br>• Transverse arch hypoplasia with isthmus inserting onto ductal arch	-	• Coarctation of the aorta<br>• Hypoplastic transverse aortic arch<br>• Common origin of brachiocephalic artery and left common carotid arteries	Surgery	88d
**27**	21	• Normal intracardiac anatomy<br>• Right aortic arch<br>• Possible aberrant left subclavian artery<br>• Left arterial duct	*Confirm arch morphology/ branching pattern*	30	• **Right aortic arch with aberrant left subclavian artery and left sided arterial duct**	-	• Right aortic arch with aberrant left subclavian artery and left sided arterial duct	CT	203d
**28**	18	• Isolated right aortic arch<br>• Smaller left sided arch cannot be ruled out<br>• Left arterial duct	*Rule out double artic arch with hypoplastic left arch*	30	• **Right aortic arch with aberrant left subclavian artery and left sided arterial duct**	-	• Right aortic arch with aberrant left subclavian artery and left sided arterial duct	Surgery	261d
**29**	29	• Coarctation of the aorta<br>• Abnormal appearance of the mitral valve	-	31	• Transverse arch hypoplasia with side-by-side insertion to ductal arch	-	• No evidence of coarctation	Echo	445d
**30**	27	• Ventricular asymmetry<br>• Great artery asymmetry<br>• Hypoplasia of the aortic arch<br>• Likely coarctation of the aorta	-	30	• Aorta significantly smaller than pulmonary artery<br>• Left aortic arch with normal branching<br>• Uniformly hypoplastic transverse arch<br>• Aortic isthmus inserts onto ductal arch	-	• Coarctation of the aorta<br>• Hypoplastic aortic arch	Surgery	6d
**31**	30	• Hypoplastic left heart syndrome<br>• Mitral and aortic atresia	-	30	• Hypoplastic ascending aorta and transverse arch<br>• Left sided aortic arch with normal branching pattern	• No evidence of pulmonary lymphangectasia	• Hypoplastic left heart syndrome<br>• Left sided aortic arch with normal branching pattern	Surgery	3d
**32**	27	• Ventricular asymmetry<br>• Great artery asymmetry<br>• Distal hypoplasia of the aortic arch<br>• Coarctation of the aorta	-	29	• Left sided aortic arch with normal branching<br>• Uniformly hypoplastic arch with proximal insertion of the isthmus to the ductal arch	-	• Coarctation of the aorta with hypoplastic aortic arch	Surgery	21d
**33**	33	• Tetralogy of Fallot<br>• Right aortic arch<br>• Right pulmonary artery not clearly seen<br>• Possible right arterial duct	*Identify pulmonary arteries*<br>*Identify arterial duct*	36	• Tetralogy of Fallot<br>• Right aortic arch with mirror image branching<br>• **Confluent branch pulmonary arteries**<br>• **Arterial duct arises from base of left subclavian artery**	-	• Tetralogy of Fallot<br>• Confluent branch pulmonary arteries<br>• Left sided arterial duct from origin of the left subclavian	Surgery	163d
**34**	27	• Atrioventricular and ventriculoarterial discordance<br>• Bilateral superior vena cavas<br>• Suspicion of coarctation of the aorta	*?Evidence of coarctation of the aorta*	33	• Ventricular and great artery asymmetry<br>• Left aortic arch with normal branching pattern<br>• **Normal insertion of the aortic isthmus with no discreet hypoplasia**<br>• Tortuous arterial duct noted	-	• Atrioventricular and ventriculoarterial discordance<br>• Bilateral superior vena cavas<br>• No evidence of coarctation of the aorta	Echo	155d
**35**	28	• Left heart hypoplasia<br>• Muscular ventricular septal defect<br>• Coarctation of the aorta	-	30	• Ventricular asymmetry with smaller but apex forming left ventricle<br>• Left aortic arch with normal branching pattern<br>• Transverse arch hypoplasia with isthmus inserting onto ductal arch	-	• Coarctation of the aorta<br>• Muscular ventricular septal defect	Surgery	9d
**36**	30	• Hypoplastic left heart syndrome<br>• Severe mitral stenosis<br>• Aortic atresia	-	30	• Hypoplastic ascending aorta and transverse arch<br>• Left aortic arch with normal branching pattern	• No evidence of pulmonary lymphangectasia	• Hypoplastic left heart syndrome<br>• Left aortic arch with normal branching pattern	Surgery	2d

**37**	28	• Tetralogy of Fallot with absent pulmonary valve<br>• Right aortic arch<br>• No arterial duct visualised	-	33	• Tetralogy of Fallot<br>• Grossly dilated pulmonary arteries<br>• Absent arterial duct<br>• Right sided arch with mirror branching<br>• **Retroaortic innominate vein**	• No segmental lung pathology	• Tetralogy of Fallot with absent pulmonary valve<br>• Absent arterial duct<br>• Right sided arch with mirror branching<br>• Retroaortic innominate vein	Surgery	25d
**38**	21	• Double outlet right ventricle with sub-pulmonary ventricular septal defect<br>• Malposed great vessels<br>• Coarctation of the aorta	-	32	• Malposed great vessels<br>• Transverse aortic arch hypoplasia<br>• **Aberrant right subclavian artery**	-	• Double outlet right ventricle with sub-pulmonary ventricular septal defect<br>• Malposed great vessels<br>• Coarctation of the aorta<br>• Aberrant right subclavian artery	Surgery	13d
**39**	26	• Right aortic arch<br>• Tortuous left arterial duct<br>• Aberrant left subclavian artery<br>• Possible small/atretic left arch	*Rule out double artic arch with hypoplastic left arch*	33	• **Right sided aortic arch with left arterial duct and aberrant left subclavian artery**<br>• **Retroaortic innominate vein**	-	• Right sided aortic arch with left arterial duct and aberrant left subclavian artery<br>• Retroaortic innominate vein	Echo	?days
**40**	22	• Single left superior vena cava<br>• Mild arterial disproportion<br>• Coarctation of the aorta cannot be excluded	*?Evidence of coarctation of the aorta*	30	• Normal situs with isolated left superior vena cava<br>• Prominent accessory hemi-azygos vein<br>• Mild ventricular and great artery disproportion<br>• **No aortic arch hypoplasia or discreet isthmal narrowing**	-	• Single left sided superior vena cava<br>• No evidence of coarctation of the aorta	Echo	272d
**41**	24	• Balanced ventricles<br>• Aortic arch smaller than ductal arch<br>• Mild isthmal hypoplasia<br>• Suspicion of coarctation of the aorta	*?Evidence of coarctation of the aorta*	31	• Mild ventricular and great artery disproportion<br>• Mild distal arch hypoplasia from left subclavian to the aortic isthmus	-	• No evidence of coarctation of the aorta	Echo	293d
**42**	30	• Multiple muscular ventricular septal defects<br>• Mild ventricular and great artery asymmetry<br>• Possible coarctation of the aorta	*?Evidence of coarctation of the aorta*	30	• Uniform aortic arch hypoplasia from left subclavian to the aortic isthmus<br>• Transverse arch hypoplasia with isthmus inserting onto ductal arch	-	• Coarctation of the aorta	Surgery	5d
**43**	28	• Aortic atresia<br>• Hypoplastic left ventricle<br>• Possible restrictive atrial septum	-	33	• Hypoplastic left ventricle<br>• Hypoplastic ascending aorta and transverse arch<br>• Left sided aortic arch, normal branching pattern	• No evidence of pulmonary lymphangectasia	• Aortic atresia<br>• Hypoplastic left ventricle<br>• Left sided aortic arch, normal branching pattern	Surgery	2d
**44**	31	• Right aortic arch<br>• Left arterial duct<br>• Branching pattern unclear	*Determine aortic branching pattern*	33	• **Right sided aortic arch with left arterial duct and aberrant left subclavian artery**	-	• Right sided aortic arch with left arterial duct and aberrant left subclavian artery	CT	254d
**45**	20	• Right aortic arch<br>• Left arterial duct<br>• Aberrant left subclavian artery	-	30	• Right sided aortic arch with left arterial duct and aberrant left subclavian artery	-	• Right sided aortic arch with left arterial duct and aberrant left subclavian artery	Echo	109d
**46**	26	• Right aortic arch<br>• Left arterial duct<br>• Aberrant left subclavian artery	-	30	• **Double aortic arch with left arch hypoplasia and left sided arterial duct**	• No segmental lung pathology/evidence of tracheal obstruction	• Double aortic arch with left arch hypoplasia and left sided arterial duct	CT	199d
**47**	28	• Right aortic arch<br>• Left arterial duct<br>• Aberrant left subclavian artery	-	33	• Right sided aortic arch with left arterial duct and aberrant left subclavian artery	-	• Right sided aortic arch with left arterial duct and aberrant left subclavian artery	Echo	146d
**48**	21	• Right aortic arch<br>• Left arterial duct<br>• Aberrant left subclavian artery	-	31	• Right sided aortic arch with left arterial duct and aberrant left subclavian artery	-	• Right sided aortic arch with left arterial duct and aberrant left subclavian artery	Echo	163d
**49**	31	• Transposition of the great arteries<br>• Sub pulmonary ventricular septal defect<br>• Possible coarctation of the aorta<br>• Poor image quality	*Visualise great vessels and aortic arch*	36	• **Aortic arch is left sided with normal branching pattern**<br>• **Hypoplastic transverse aortic arch**<br>• **Aortic isthmus displaced proximally with superior insertion onto arterial duct**	-	• Transposition of the great arteries<br>• Sub pulmonary ventricular septal defect<br>• Aortic arch hypoplasia with coarctation of the aorta	Surgery	9d

**50**	20	• Right aortic arch<br>• Left arterial duct<br>• Mirror image branching pattern	-	29	• **Right sided aortic arch with left arterial duct and aberrant left subclavian artery**	-	• Right sided aortic arch with left arterial duct and aberrant left subclavian artery	Echo	18d
**51**	22	• Right aortic arch<br>• Left arterial duct<br>• Aberrant left subclavian artery	-	31	• Right sided aortic arch with left arterial duct and aberrant left subclavian artery	-	• Right sided aortic arch with left arterial duct and aberrant left subclavian artery	Echo	15d
**52**	28	• Right aortic arch<br>• Left arterial duct<br>• Mirror image branching pattern	-	33	• **Double aortic arch with left arch hypoplasia and left sided arterial duct**	• No segmental lung pathology/evidence of tracheal obstruction	• Double aortic arch with left arch hypoplasia	Echo	134d
**53**	28	• Right aortic arch<br>• Left arterial duct<br>• Unclear branching pattern	*Determine aortic branching pattern*	29	• **Right sided aortic arch with left arterial duct and aberrant left subclavian artery**	-	• Right sided aortic arch with left arterial duct and aberrant left subclavian artery	Echo	52d
**54**	27	• Possible coarctation of the aorta<br>• Ventricular septal defect<br>• Transverse arch hypoplasia	*?Evidence of coarctation of the aorta*	31	• **Mild ventricular and great artery disproportion with mild transverse arch hypoplasia only**	-	• No coarctation of the aorta<br>• **Partial anomalous pulmonary venous drainage (left upper pulmonary vein to innominate vein)**	Echo	7d
**55**	30	• Mitral atresia<br>• Multiple ventricular defects<br>• Double outlet right ventricle<br>• Possible coarctation of the aorta<br>• Moderate tricuspid valve regurgitation	-	35	• Double outlet right ventricle<br>• Pulmonary artery anterior to aorta<br>• Dominant right ventricle with severely hypoplastic left ventricle<br>• Great artery disproportion with severe hypoplasia of the transverse arch	-	• Double outlet right ventricle<br>• Severe mitral stenosis with left ventricle<br>• Subaortic stenosis<br>• Multiple ventricular septal defects<br>• Coarctation of the aorta with severe arch hypoplasia	Surgery	5d
**56**	24	• Possible double aortic arch with dominant right and left arterial duct<br>• Difficult to visualised if left arch is complete	*Confirm double aortic arch*	30	• **Double aortic arch with left arch hypoplasia and left sided arterial duct**	• No segmental lung pathology/evidence of tracheal obstruction	• Double aortic arch with left arch hypoplasia	CT	58d
**57**	21	• Right aortic arch<br>• Left arterial duct<br>• Indeterminate arch branching pattern	*Determine aortic branching pattern*	29	• **Right sided aortic arch with left arterial duct and aberrant left subclavian artery**	-	• Right sided aortic arch with left arterial duct and aberrant left subclavian artery	Echo	52d
**58**	29	• Bilateral superior vena cavas<br>• Interrupted inferior vena cava with azygous continuity to left superior vena cava<br>• High suspicion of coarctation of the aorta	-	32	• Bilateral superior cavas<br>• Interrupted inferior vena cava with hemi-azygos continuation to left superior vena cava<br>• Transverse arch hypoplasia, particularly aortic isthmus	-	• Discrete juxta-ductal coarctation of the aorta<br>• Bilateral superior vena cavas<br>• Interrupted inferior vena cava with hemi-azygos continuation to left superior vena cava	Surgery	7d
**59**	30	• Pulmonary atresia<br>• Ventricular septal defect<br>• Unable to visualise branch pulmonary arteries<br>• Collaterals suspected	*Confirm pulmonary arterial supply*	35	• **Hypoplastic branch pulmonary arteries**<br>• **Major aortopulmonary collaterals visualised from descending aorta**	• Thymus present	• Pulmonary atresia<br>• Ventricular septal defect<br>• Confluent hypoplastic pulmonary arteries<br>• Major aortopulmonary collateral arteries	CT	33d
**60**	20	• Right aortic arch<br>• Left arterial duct<br>• Aberrant left subclavian artery	-	32	• Right sided aortic arch with left arterial duct and aberrant left subclavian artery	-	• Right sided aortic arch with left arterial duct and aberrant left subclavian artery	Echo	109d
**61**	32	• Multiple pulmonary arteriovenous malformations in left lung<br>• Dilated vessel entering left atrium	*Determine left sided pulmonary arterial and venous supply*	34	• **Complex arteriovenous malformation from the left pulmonary artery, multiple branches extending throughout most of the left lung**<br>• **Large pulmonary venous vessel to left atrium**<br>• **No additional collaterals to either the left lower lobe or to the right lung**	• Brain imaging<br>• Lung imaging	• Poor ventricular function<br>• Died at 1 hours of age – no cross-sectional imaging<br>• No post-mortem	-	-
**62**	27	• Right aortic arch<br>• Right arterial duct<br>• Aneurysm of the ventricular septum	-	31	• Right sided aortic with mirror image branching<br>• Right sided arterial duct	-	• Right aortic arch<br>• Right sided arterial duct	Echo	2d

**63**	21	• Right aortic arch<br>• Left arterial duct<br>• Aberrant left subclavian artery	-	33	• Right sided aortic arch with left arterial duct and aberrant left subclavian artery	-	• Right sided aortic arch with left arterial duct and aberrant left subclavian artery	Echo	8d
**64**	27	• Hypoplastic aortic arch<br>• Coarctation of the aorta<br>• Ventricular septal defect	-	30	• Mild great artery disproportion with transverse arch and isthmal hypoplasia	-	• Doubly committed ventricular septal defect<br>• Aortic arch hypoplasia<br>• Coarctation of the aorta	Surgery	7d
**65**	24	• Right aortic arch<br>• Left arterial duct<br>• Difficult to be confident about branching pattern	*Determine aortic branching pattern*	31	• **Right sided aortic arch with left arterial duct and aberrant left subclavian artery**	-	• Right sided aortic arch with left arterial duct and aberrant left subclavian artery	CT	151d
**66**	23	• Right aortic arch<br>• Left arterial duct<br>• Origin of left subclavian could not be confirmed	*Determine aortic branching pattern*	32	• **Right sided aortic arch with left arterial duct and aberrant left subclavian artery**	-	• Right sided aortic arch with left arterial duct and aberrant left subclavian artery	Echo	34d
**67**	27	• Large ventricular septal defect<br>• Hypoplastic aortic arch<br>• Coarctation of the aorta	-	31	• Great vessel disproportion<br>• Transverse and distal aortic hypoplasia	-	• Large ventricular septal defect<br>• Hypoplastic transverse arch, maximal narrowing at isthmus<br>• Coarctation of the aorta	Surgery	9d
**68**	29	• Complete atrioventricular septal defect<br>• Ventricular disproportion (left ventricle smaller than the right ventricle)<br>• Aortic arch appears to taper distally raising suspicion of aortic coarctation	*?Evidence of coarctation of the aorta*	32	• **Significant aortic arch hypoplasia, particularly distal arch and isthmus**	-	• Unbalanced atrioventricular septal defect<br>• Aortic arch hypoplasia<br>• Coarctation of the aorta<br>• Intrauterine growth restriction	Surgery	7d
**69**	28	• Left ventricle smaller than right<br>• Disproportion of the great arteries<br>• Slender transverse arch<br>• Suspected coarctation of the aorta	*?Evidence of coarctation of the aorta*	31	• **Great vessel disproportion**<br>• **Transverse and distal aortic hypoplasia**	-	• Slender transverse arch and isthmus, no evidence coarctation of the aorta	Echo	146d
**70**	33	• Perimembranous ventricular septal defect<br>• Right aortic arch<br>• Suspicion of aorto-pulmonary window	*Confirm great artery anatomy*	34	• **Right aortic arch with mirror image branching pattern**<br>• **Large proximal aortopulmonary connection proximal to origin of brachiocephalic artery**	-	• Large proximal aortopulmonary connection distal to arterial valve but proximal to origin of brachiocephalic artery	Surgery	19d
**71**	33	• Tetralogy of Fallot<br>• Right sided aortic arch<br>• ?Confluent branch pulmonary arteries<br>• Unable to identify arterial duct<br>• Poor echo windows	*Arterial duct anatomy*<br>*Confirm pulmonary arterial supply*	34	• **Good sized, confluent main and branch pulmonary arteries**<br>• **Right aortic arch with mirror image branching**<br>• **No detectable arterial duct**<br>• **No detectable aortopulmonary collaterals**	• Thymus present	• Tetralogy of Fallot<br>• Right sided aortic arch<br>• Confluent branch pulmonary arteries<br>• No arterial duct	Surgery	157d
**72**	31	• Right aortic arch<br>• Left arterial duct<br>• Origin of left subclavian could not visualised	*Determine aortic branching pattern*	32	• **Right sided aortic arch with left arterial duct and aberrant left subclavian artery**	-	• Right sided aortic arch with left arterial duct and aberrant left subclavian artery	Echo	16d
**73**	21	• Right aortic arch<br>• Left arterial duct<br>• Mirror image branching pattern	-	30	• **Right sided aortic arch with left arterial duct and aberrant left subclavian artery**	-	• Right sided aortic arch with left arterial duct and aberrant left subclavian artery	Echo	22d
**74**	28	• Normal cardiac connections<br>• Dilated distal ascending aorta<br>• Dilated transverse arch	-	31	• Isolated dilatation of the proximal ascending aorta and to transverse aortic arch	• Vascular imaging of descending aorta<br>• Cerebral imaging	• Mildly dilated ascending aorta, aortic sinuses, sinotubular junction and ascending aorta	Echo	128d
**75**	31	• Hypoplastic left heart syndrome<br>• Normal aortic branching pattern	-	31	• Hypoplastic ascending aorta and transverse arch<br>• Normal aortic branching pattern	• No evidence of pulmonary lymphangectasia	• Hypoplastic left heart syndrome<br>• Left aortic arch with normal branching pattern	Surgery	3d
**76**	27	• Hypoplastic left heart syndrome	-	34	• Hypoplastic ascending aorta and transverse arch<br>• **Normal aortic branching pattern**	• No evidence of pulmonary lymphangectasia	• Hypoplastic left heart syndrome<br>• Left aortic arch with normal branching pattern	Surgery	3d

**77**	29	• Bilateral superior vena cavas<br>• Aortic arch unable to be clearly visualised<br>• Unable to exclude coarctation of the aorta	-	34	• Bilateral superior vena cavas<br>• **Mild ventricular asymmetry**<br>• **No frank aortic arch or isthmal hypoplasia**	-	• Bilateral superior vena cavas<br>• Bicuspid aortic valve<br>• No coarctation of the aorta	Echo	27d
**78**	32	• Hypoplastic left heart syndrome<br>• Pulmonary veins cannot be seen to drain normally to the left atrium<br>• Confluence behind left atrial mass<br>• Possible anomalous pulmonary venous drainage	*Determine pulmonary venous drainage*	33	• Hypoplastic left heart syndrome<br>• **Total anomalous pulmonary venous drainage via ascending vein (supracardiac)**<br>• **Potential compression of ascending vein between left pulmonary artery and arterial duct**	• Pulmonary lymphangectasia *("nutmeg lung")*	• Compassionate care only after birth<br>• Died 1d age<br>• No post-mortem	-	-
**79**	29	• Ventricular disproportion<br>• Aortic isthmus within normal limits<br>• Cannot exclude coarctation of the aorta	*?Evidence of coarctation of the aorta*	33	• **Normal aortic arch geometry with no evidence of discreet arch or isthmus hypoplasia**	-	• Mild ventricular asymmetry<br>• No evidence of coarctation of the aorta	Echo	69d
**80**	20	• Double aortic arch (right arch dominant)<br>• Left arterial duct	-	30	• Double aortic arch with dominant right sided arch and smaller left arch.<br>• **Retroaortic innominate vein**	• No segmental lung pathology/evidence of tracheal obstruction	• Double aortic arch with dominant right sided arch and smaller left arch.<br>• Retroaortic innominate vein	Echo	13d
**81**	29	• Coarctation of the aorta<br>• Aorta appears significantly smaller than the pulmonary artery with a long segment of the arch appearing hypoplastic	-	30	• Slender transverse arch with proximal insertion of the isthmus with respect to the arterial duct<br>• Bilateral superior vena cavas	-	• Bicuspid aortic valve<br>• Coarctation of the aorta	Surgery	7d
**82**	23	• Right aortic arch<br>• Left arterial duct<br>• Mirror image branching pattern	-	32	• **Right sided aortic arch with left arterial duct and aberrant left subclavian artery**	-	• Right sided aortic arch with left arterial duct and aberrant left subclavian artery	Echo	14d
**83**	32	• Common atrium<br>• Double outlet right ventricle<br>• Mitral and aortic atresia<br>• Hypoplastic left ventricle<br>• Aberrant right subclavian artery	-	32	• Double outlet right ventricle with mitral atresia and severe hypoplasia of the left ventricle<br>• Arch hypoplasia with aberrant origin of the right subclavian artery	• No evidence of heterotaxy syndrome	• Double outlet right ventricle<br>• Hypoplasia of the left ventricle<br>• Left aortic arch with aberrant right subclavian artery	Surgery	7d
**84**	22	• Unbalanced four chamber view<br>• Great artery asymmetry<br>• Aortic and ductal arch asymmetry<br>• Distal aortic arch hypoplasia<br>• Coarctation of the aorta	-	32	• Hypoplastic ascending aorta and transverse aortic arch with isthmus inserting onto ductal arch	-	• Coarctation of the aorta<br>• Hypoplastic aortic arch	Surgery	9d
**85**	28	• Normal cardiac situs<br>• Heart rotated to the left<br>• Overriding aorta<br>• Pulmonary atresia with possible disconnected pulmonary arteries	*Pulmonary arterial supply*	32	• Pulmonary atresia with ventricular septal defect<br>• **Disconnected, non-confluent left and right branch pulmonary arteries, each supplied by a single ipsilateral collateral**<br>• **Left collateral from origin of left common carotid**<br>• **Right collateral from underside of distal aortic arch**<br>• **Right sided aortic arch with aberrant left subclavian artery**	• No visible thymus tissue	• Pulmonary atresia with ventricular septal defect<br>• Bilateral collaterals supplying left and right pulmonary arteries<br>• Left collateral from origin of left common carotid<br>• Right collateral from underside of distal aortic arch<br>• Right sided aortic arch with aberrant left subclavian artery<br>• Absent thymus<br>• Microdeletion on chromosome 4	Surgery	9d

GA *= gestational age (weeks)*
Bold text = key MRI findings
Bold underlined text = new findings
